# Microbiota as a New Target in Cancer Pathogenesis and Treatment

**DOI:** 10.7759/cureus.47072

**Published:** 2023-10-15

**Authors:** Abeer S Algrafi, Aisha A Jamal, Dana M Ismaeel

**Affiliations:** 1 Internal Medicine, College of Medicine, Taibah University, Madinah, SAU; 2 General Practice, College of Medicine, Taibah University, Madinah, SAU

**Keywords:** chemo-resistance, immunotherapy, cancer immunity, microbiome, microbiota

## Abstract

The microbial ecosystem of humans is an integral part of human health and disease. A significant percentage of tumors worldwide are thought to be microbially induced. The relationship between cancer and microbes is complex. In this article review, we aim to give an overview of human microbiota and its role in carcinogenesis, emphasize the relation between microbiota and cancer immunity, and highlight its role in the future of cancer therapy. The term microbiota refers to the collection of microorganisms that are located in an individual, whereas the total genome of these microorganisms is referred to as the microbiome. The microbiota in humans has many physiological functions. The microbiota within the gut lumen has a profound effect on the local and systemic immune system. The immune system can change the gut microbiota. Microbiota may induce carcinogenesis by several mechanisms. It also affects tumor progression. Thus, microbiota modulation may aid in the prevention and treatment of cancer. Intentionally introducing microorganisms into the oncological patient is assumed to mobilize the immune system to become able to, at least, limit the development of cancer. Microbes are used as vectors which are carriers of particular antineoplastic agents that reduce the side effects of chemotherapy. Inflammation and tumor microenvironment play an essential role in promoting chemo-resistance. There is now considerable evidence, both in humans as well as in laboratory animals, that the commensal microbiota has important effects on carcinogenesis, tumor growth, and therapy response.

## Introduction and background

The microbial ecosystem of humans is an integral part of human health and disease. These microbes reside on the epithelial surface of the body's barrier. On the surface of the epithelial barrier of the body, there are many microorganisms such as bacteria, viruses, archaea, fungi, and protozoa that make up the human microbiome [1].

Twenty percent of tumors worldwide are thought to be microbially induced [2]. Although cancer is generally considered a disease of the genetics of the host and environmental factors, the relationship between cancer and microbes is a complex and ongoing area of research [3].

Cancer is the world's second-largest cause of death, with an estimated 9.6 million deaths in 2018. Globally, cancer causes about one in six deaths [4]. In Saudi Arabia, the prevalence of cancer in five years is 70,819 cases according to Global Cancer Observatory. In 2018, there were an estimated 24,485 new cases, with breast cancer accounting for 14.8% and colorectal cancer accounting for 14.6% of the total [5].

Cancer remains one of the 21st century's biggest challenges. The increasing number of cases is not accompanied by adequate progress in therapy. The standard treatment methods often do not result in the expected effects. Hence, finding new, more effective treatments is extremely crucial [6].

Chemotherapeutic agent resistance continues to be the defining characteristic of cancer therapy which involves various cell-intrinsic mechanisms. Chemo-resistance results in undesirable outcomes including recurrence, metastasis, and diminished patient survival [7].

Recent studies have introduced the potential effects of the bacterial ecosystem on chemotherapeutic drugs’ effectiveness, metabolism, and toxicity [8]. It was proposed that the microbiota have an important positive role in modulating the efficacy of a variety of anti-cancer therapeutic interventions [9].

In this article review, we aim to give an overview of human microbiota and the microbiome, demonstrate the role of microbiota in carcinogenesis, emphasize the relation between microbiota and cancer immunity, and highlight the possible role of microbiota in the future of cancer therapy.

## Review

Human microbiota

The term microbiota applies to the collection of microorganisms including bacteria, viruses, fungi, archaea, and protozoa that are present in the body, while the microbiome refers to their total genome [10]. Microorganisms are present in various areas of the human body, on the external and internal surfaces, including the gastrointestinal tract, lungs, oral and genital mucosa, skin, saliva, bladder, and conjunctiva [11].

The human microbiota is mainly colonized by Bacteroidetes, Firmicutes, Actinobacteria, Proteobacteria, Cyanobacteria, and Fusobacteria [11]. The microbiota of an organism is a highly variable and compartmentalized ecosystem. Diversity levels among body sites vary, with the mouth and gut harboring the most diverse communities [10].

The development of microbiota has been highlighted in several studies that have suggested factors influencing microbial development and diversity. The method of delivery is important; 20 minutes after birth, babies that were delivered vaginally display vaginal microbiota that closely resembles that of the mother. However, the infants who were delivered by a Cesarean section own microorganisms naturally found on human skin [12].

As early as the first year of life, the infant’s gastrointestinal tract microbiome begins to be more similar to that of an adult. The development of microbiota continues throughout the first several years of life [13]. Other research has underlined the impact of food intake in influencing the body's microbial composition, demonstrating that dietary changes correspond to changes in the gut microbiota and enrichment of their genes. Hence, introducing an adult’s diet to an infant has been shown to enhance genes in the microbiome associated with vitamin biosynthesis and polysaccharide digestion [14].

The human microbiota has many physiological functions in the body. The gut microbiome, for example, performs various essential functions such as food hydrolysis, vitamin synthesis, pathogen colonization management, and defense against systemic infections. The gut microbiota also plays a crucial role in immune system development [9].

Microbiota and Microbiome

There is some confusion between the term microbiota (the human-related microbial taxa) and the term microbiome (the set of those microorganisms and their genes). Typically, those two terms are used interchangeably [10]. The microbiome can be described as ’the microbiota genes and genomes, along with the products of microbiota and the host environments’, including plasmid deoxyribonucleic acid (DNA) and viruses, fungi, and archaea, though they are not commonly observed [15]. The human microbiota consists of 10-100 trillion symbiotic microbial cells present in each individual, usually intestinal bacterial cells; however, the human microbiome refers to the genes that the cells contain [10]. Simply put, the microbiome is the specific genomes of microbes within a population, whereas the term microbiota refers to the aggregate of microbes [15].

Tumor Microbiota

Studies have shown that when cancer develops, the microbial composition of many parts of the body changes. There are clear microbial changes in the tumor environment, indicating a role for the microbiome in promoting carcinogenesis. A hypoxic tumor microenvironment is proposed to increase the growth rate of anaerobic bacteria and facultative anaerobic bacteria such as Clostridia. In addition, necrotic tumor areas can cause the release of chemotactic compounds and attract bacterial invasion [16].

The increased permeability of blood vessels allows bacteria to enter the tumor environment. The absence or even reduction of immune cells may allow tumor growth [17]. Mucosal tumors have close communication with bacteria and hence are vulnerable to being influenced by the microbiome. Bacteria enter the necrotic microenvironment of mucosal cavities, adjacent body fluids, or elsewhere and can invade the cell, which weakens immune surveillance [16].

Microbial composition changes are observed in different cancers, such as colorectal, pancreatic, lung, breast, endometrial, and prostatic [16]. The colo-microbiome (CRC) has been widely studied since it is subjected to a wide variety of bacterial species in the gut. In numerous studies, Fusobacterium nucleatum, in particular, was found to be nourished in colonic tumor tissue, even though it remains unclear if it is a causal factor or a spectator of any other process. These bacteria are further correlated with histological grade, chemotherapy resistance, cytosine-phosphodiester-guanine (CpG) island methylation phenotype status, cluster of differentiation 3+ thymus cells (CD3+ T-cell) infiltration, and deprived survival rates [18].

Another organism is Campylobacter jejuni, which was found to promote the growth and enlargement of colorectal tumors in mice, and induces changes in microbial composition and transcriptomic responses, a process dependent on cytolethal distending toxin (CDT) production [19]. Infection with Streptococcus gallolyticus in the form of endocarditis or even bacteremia is correlated to colorectal cancer. In addition, this class is also enriched in the tissue of the tumor itself [20].

In pancreatic cancer, using fluorescent-labeled microbes, bacteria were observed to dislocate to the pancreas from the gut. The microbiome is capable of reprogramming the pancreatic immune microenvironment, with microbiome resection allowing increased CD4+ and CD8+ T cells and reduced myeloid suppressor cells. In mice, microbiome recolonization helps restore an immunosuppressive environment in the pancreatic tumor, however, it may not restore the typical microbiome of the gut, since oral gavage may trigger a larger number of bacteria and bacterial translocation [21].

Airway epithelium has a distinct microbiome that could be altered in many disease states including chronic obstructive pulmonary disease, cystic fibrosis, asthma, and lung cancer. Streptococcus and Veillonella enrichment in lung cancer brushings have been associated with upregulation of phosphoinositide 3-kinases (PI3K); pPI3Ks, also known as phosphatidylinositol 3-kinases and extracellular signal-regulated kinase (ERK) pathways, are an enzyme group involved in cellular processes such cell growth, proliferation, differentiation, motility, survival, and intracellular trafficking that are proposed to have a role in promoting growth, migration, treatment resistance, and metastasis in most lung cancers [22,23]. Haemophilus influenza has been introduced to promote carcinogenesis through interleukin (IL)-17C and neutrophil infiltration [24].

Human breast cancer tissue has reportedly been found to have a distinct microbiome from surrounding skin or other tissues. The microbial composition of different breast cancer subtypes has been found to vary. Both triple-positive and triple-negative tumor types showed distinct patterns [25].

The gut microbiome can play a role in the development of breast cancer as it affects both estrogen-dependent and non-estrogen-dependent processes. Dysbiosis, or an imbalance in the composition of the gut microbiota, can alter the production of metabolites that may promote the growth of breast cancer and disrupt estrogen metabolism. Some studies have investigated the link between gut microbiome diversity and levels of estrogen, and have suggested that certain types of bacteria and a higher diversity may be associated with an increased risk of breast cancer [26].

Microbial Dysbiosis

The gut microbiota is characterized by temporal constancy and resilience, which refers to the capacity to recover after disturbances. Constant modification may occur when environmental changes exceed the microbiota's capacity for resilience which is defined as dysbiosis. Dysbiosis contributes to the non-control of pathogenic microbes and an inflammatory or dysregulated immune response to microbes [27].

Dysbiosis is related to many diseases, including metabolic diseases, inflammatory diseases, and cancers. Microbial dysbiosis has been proposed to contribute to cancer pathogenesis, progression, and therapy outcome due to the ability of the microbiota to modulate host inflammation and immune responses and to metabolize drugs and xenobiotics [28].

Microbiota Contribution to Carcinogenesis

The International Agency for Cancer Research (IACR) classified only 10 of all microbes on Earth, estimated at 3.7 × 1030, to be oncogenic to humans [29]. The pathogens described are Helicobacter pylori associated with gastric cancer, Epstein-Barr virus associated with gastric cancer and lymphoma, hepatitis B and C viruses associated with liver cancer, Aspergillus spp. associated with liver cancer, Opisthorchis viverrine associated with bile duct cancer, Clonorchis sinensis associated with bile duct cancer, Fusobacterium nucleatum associated with colorectal cancer, Schistosoma haematobium associated with bladder cancer, human papillomavirus associated with cervical cancer, and Kaposi’s sarcoma herpes virus associated with Kaposi’s sarcoma [30]. Although most of these cancer-causing microbes colonize big proportions of the global population, just a small percentage of infected people develop cancer, since the host and microbial genotypes regulate cancer susceptibility [3].

Microbiota forms to induce carcinogenesis can be divided into three main classes. First, changing the balance of cell proliferation and destruction. The second mechanism is by directing the functioning of the immune system. Finally, by modulating the metabolism of the factors produced by the host, ingested nutrients, and medications [3].

There are various ways that microbes might affect the development and spread of cancer. First, toxins produced by microbes can damage DNA directly by the produced toxins or indirectly by the reactive oxygen and nitrogen radicals produced by the host cells. When DNA damage exceeds the capacity of the host cell to repair it, apoptosis or cancer-causing mutations occur in the cell. Second, changes in the β-catenin signaling pathway are a major target of microbes related to cancer. Many microbes bind to epithelial cadherin (E-cadherin) in epithelial cells which leads to altered polarity or impaired barrier function and stimulates β-catenin. Certain microbes insert effectors, such as cytotoxin-associated gene A (CagA), which activate β-catenin signaling, resulting in uncontrolled cell proliferation and loss of cell polarity. Furthermore, inflammatory processes in developing tumors include a break of the membrane barrier. This loss of barriers for the host by microbe involves activation of receptors for pattern recognition as well as their signaling cascades that ultimately lead to activation of several cytokines and recruitment of the inflammatory cells which is induced by nuclear factor kappa B (NF-κB) and signal transducer and transcription activator (STAT3) signaling that lead to inflammation-associated disease. Indeed, NF-κB, especially in immune cells, is a master regulator of cytokine expression whose action on epithelial and cancer cells promotes survival and proliferation and chemokine expression [3].

While commensal bacteria may play an essential role in the carcinogenesis of animals and humans, the International Agency for Research on Cancer described Helicobacter pylori as the only class I human carcinogen bacterial species because of its association with stomach cancer and lymphoma [31]. It was found that H. pylori-induced stomach cancer involves multi-decade bacterium contact, in addition to an early inflammatory response. H. pylori affects the host epithelial tissues in several ways which could promote tumorigenesis, by induction of proliferation, the inflammatory response and apoptosis. One of the mechanisms is the production of IL-1β by dendritic cells (DCs) infected with H. pylori, which damage and atrophy the epithelium, decrease in acid secretion, and induce intestinal metaplasia [32]. Genetic changes of Kirsten rat sarcoma (K-ras) and tumor protein 53 (p53) have been seen commonly in gastric adenocarcinoma, implicating that stomach cancer pathogenesis depends predominantly on the pathogenic inflammatory response [32].

**Figure 1:**
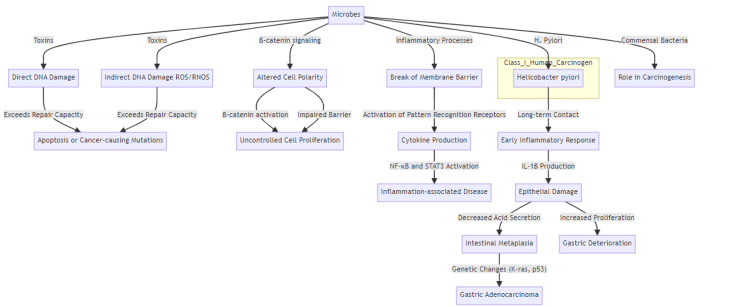
Microbiota contribution to carcinogenesis

However, in people with severe atrophic gastritis, H. pylori is not detected anymore, even if serological tests indicate previous infection [33]. Such findings suggest an alternative proposal for H. pylori-induced cancers in which gastric deterioration motivated by long-lasting H. pylori host contact induces dysbiosis (Figure 1) [34].

Microbiota and cancer immunity

Microbiota within the gut lumen has a profound effect on the local immune system in the gut mucosa, in the drainage of mesenteric lymph nodes, and systemically. The induction, development, and activity of the host immune system are fundamentally influenced by the microbiota. In exchange, the immune system has largely developed as a way for the host to sustain its mutual association with these extremely diverse and constantly changing microbiotas. Goblet cells form a thick protective layer of mucus that covers the mucosa. In the lamina propria, the plasma cells secrete immunoglobulin A (IgA) into the gut lumen. Paneth cells produce an amount of anti-microbial peptides, which have an enhanced activity in response to microbiota-related signaling from the immune system [15].

Bacterial products or the bacterium itself can trigger DCs that promote the activation of naive T cells into effector T cells, helper T cells (Th17), or regulatory T cells (Tregs) in the lymph nodes. Then T cells may reach the circulation or move back to the gut. Different metabolites or bacterial products may modify the DC in a way that induces them to shift towards a phenotype Treg versus Th17. Tregs exert their role when IL-10 is secreted, producing local anti-inflammatory cytokines. Meanwhile, Th17 cells produce IL-17, which can increase the production of anti-microbial peptides in Paneth cells and can work within the enlistment of polymorphonuclear neutrophils (PMNs) from the circulation system. A few bacterial metabolites may enter the systemic circulation and may directly alter the immune system [15].

Several mechanisms that make the intestinal microbiota important for the activation and function of mucosal immunity have been described. For instance, the recruitment of IgA-secreting plasma cells and activated T cells and the complete development of the gut-associated lymphoid tissues (GALTs) of mucosal surfaces were found to require microbiota-derived signals that act on DCs and epithelial cells after birth [35].

In vertebrates, many microbiota products, and pathogens acting mainly on the innate receptors of toll-like receptor (TLR) and nucleotide-binding oligomerization domain-like receptors (NOD-like receptors), influence the immunity of the barrier through pro-inflammatory and anti-inflammatory mechanisms. The function of IL-1 receptors and TLRs in regulating the environment of gut microbiota was demonstrated in mice lacking specific adapter molecules myeloid differentiation 88 (MyD88), where the expression of microbiota-regulated genes is changed [36]. MyD88 signaling is necessary for antimicrobial gene epithelial expression, such as regenerating islet-derived protein (Reg3β), and MyD88 deficiency has been shown to alter bacterial structure and diversity [36].

In addition to triggering pro-inflammatory immune circuits, microbes also exploit or evoke immunosuppressive responses. To avoid destruction, a microbe may take advantage of pre-existing immunosuppression or trigger immune-dampening responses. As seen with advanced human immune-deficiency virus (HIV) infection, the chronic immunosuppressed patient raises the risk for many cancers, particularly virally related malignancies. Microbial-caused immunosuppression may also contribute to impaired antitumor immunity. Lots of the recent cancer-directed immunotherapies focus on inducing tumor immune response [37].

Cancer of the colon-associated bacterium F. nucleatum can specifically inhibit antitumor immunity by involving certain T cells, immunoglobulin receptor cells, and natural killer cells, and by preventing its ability to damage tumor cells [38]. Two mechanisms limit the response of the host to commensals, likely contributing to the host their peaceful and symbiotic coexistence. First, a bacterial fermentation product called short-chain fatty acids (SCFAs), are found to modulate mucosal immunity in the intestine; by sending a signal through guanine nucleotide-binding proteins (G-protein) coupled receptors, SCFAs stimulate enterocyte production of IL-18 and they act directly on the Treg, regulating its size and role [39].

Also, Bacteroides fragilis, a typical constituent of the gut microbiota, drives the differentiation of IL-10-secreting Treg cells by signaling via its capsular polysaccharide [35]. Bacteroides fragilis also were found to protect mice from infection with Helicobacter hepaticus and colitis induced by trinitrobenzene sulfonic acid (TNBS) [15].

The segmented filamentous bacteria (SFB) can improve the immune response of mucosal tissues. SFB are bacteria that can not be cultured present at weaning in the ileum of mice. This bacterial species induces the immune response of the gut mucosa after birth [39]. In the absence of SFB, mice were found to possess reduced IgA titers and decreased numbers of helper T-cells (especially Th17 cells). Also, they had a weak response to intestinal pathogenic bacteria such as Salmonella and Citrobacter rodentium [15].

Microbiota and cancer thereby

The treatment of cancer has been a very important unsolved problem over the last two centuries. Several methods are currently used in the treatment of cancer. They can fall into local or systemic therapeutic methods, the combination of both types of methods usually is practiced. Local treatment includes oncological surgery, the most important, and radiotherapy. This includes tumor irradiation, which results in compromised proliferation and metabolic functions [6].

Systemic therapy involves chemotherapy, biological therapy, and hormone therapy. Chemotherapy involves agents that interfere with cell division of all highly dividing cells. Hence, it affects and destroys not only cancer cells but also normal body cells [6]. Biological treatment involves directing monoclonal antibodies against the exact antigens of cancer cells and using substances to block the metabolic pathways of the cancer cell. Hormone therapy is only used in neoplasms where effective hormone receptors are expressed [40].

Anticancer treatment even includes the usage of modified DCs or tumor cells in vaccines. Many years ago, the use of microorganisms immunizations was already used as a treatment to activate the immune response of the individual; nevertheless, this type of therapy is still under-examined [41]. The origin of microbial use in cancer therapy started when Dr. William Coley, the father of immunotherapy, created a mixture of microbes that successfully treated specific types of cancer. This was the first time microbial therapy was used in cancer treatment [6].

Microbiota and Prevention of Cancer

The microbiome appears to have a role in tumorigenesis, and microbiota modulation may aid in the prevention of cancer. There is also a possibility for genetically modifying microbes and for the administration of antibiotics, genotoxins, or medications directing bacterial-induced inflammation, to inhibit tumorigenesis [16].

One study about colonic tumorigenesis on laboratory mice revealed that oral administration of a Lactobacillus helveticus-containing probiotic supplement may reduce the number of T lymphocytes producing IL-17 and control hyperplasia and carcinogenesis maybe by modulating the gut microbiota [16]. Another study showed that in patients who have colonic polyps or neoplasms, the administration of synbiotics has the potential to enhance the function of epithelial barriers, modulate microbiota composition in feces and decrease the proliferation of colon cells [42].

The National Institute of Health compared the microbiota of the 21 common laboratory mouse strains to that of a similarly related wild strain relative. When natural “wild-type” microbiota was implanted in the laboratory mice, they showed higher resistance to carcinogens and colonic carcinogenesis that is induced by inflammation. Altering the microbiota in the precancerous stage could offer a way to avoid the initiation of cancer [43].

The Impact of Microbiota on Immunotherapy

There are considerable pieces of evidence that the human microbiome can influence immunotherapy effectiveness. The common use of some types of immunotherapy and the emergence of checkpoint inhibitors revealed a new relationship between microbiome and cancer therapy. Abnormal gut microbiome and antibiotic treatment are associated with resistance among patients with progressive cancers treated by immunotherapy [16].

The response to an immune checkpoint inhibitor, anti-programmed cell death protein/ligand 1 (anti-PD-1/PD-L1), or anti-cytotoxic T-lymphocyte-associated protein-4 (anti-CTLA-4), can be significantly changed by the constituent gut microbiota. Regarding PD-1/PD-L1 blockade, noticeable variations in treatment response were seen in mice with different microorganisms. Studies of the intestinal microbiota found that Bifidobacterium species are more abundant in mice with the slow development of the tumor and positive anti-PD-1 responses [44].

A positive result of a mouse possessing desirable microbiota may be passed to another mouse via co-housing or fecal microbiota transplantation (FMT). Additionally, oral probiotics containing Bifidobacterium were given to mice with unfavorable gut microbiota, which then, in turn, enhanced the anti-tumor PD-L1 blockade effectiveness. That whole influence comes mainly from improving the maturation of DC, thus increasing the tumor-specific activity of T cells + CD8 [44].

In metastatic melanoma patients, anti-PD-1 treatment was also affected by intestinal microbiota. In patients with PD-1 treatment, the diversity in gut microbiota was significantly increased. And some of the microbes, including Ruminococcaceae, Faecalibacterium, and Clostridiales were comparatively more common. Patients who did not respond to PD-1 therapy had less bacterial variety and a greater amount of Bacteroidales [45].

Microbiota as Anticancer Agent

The introduction of microorganisms into the body results in the activation of immune mechanisms, which, in turn, leads to a rise in the amount of innate immune cells such as NK cells, macrophages, and neutrophils. It leads also to the stimulation of the acquired immune system i.e. B lymphocyte and T lymphocyte, and the reinforcement of synthesis of pro-inflammatory cytokines [6]. It is assumed that the deliberate introduction of microbes into the cancer patient mobilizes the immune system to at least become able to restrict tumor growth. Microbes can contribute indirectly to the regression of cancer, particularly in patients who did not respond to other widely used therapies [6].

The safety of the microbes used is very necessary as the goal is to treat the tumor, not to affect the patient’s health by harming one with a microorganism. Different types of methods are undertaken to maintain the safety of preparations such as attenuation which deprives the microbes of their pathogenicity [46]. In addition, the presence of bacterial secretion products as toxins in the tumor environment appears to have therapeutic potential, as they may induce a destructive impact on the tumor [6].

The consumption of sporangial bacteria represents another method used in the experiments with Clostridium novyi, which can live under unfavorable environmental conditions. This micro-organism favors anaerobic environments that are present in the tumor. Rather than spreading throughout the body, the bacteria are guided only to the tumor area, where they have an ideal growth environment. This microbial property protects the patient from the occurrence of serious infections [46]. The use of bacterial microbial preparations as anticancer treatment complements conventional therapy and improves the likelihood of full recovery for the patients. The bacteria can be used for therapeutic purposes in different forms [6].

Mycobacterium bovis: there are associations between tuberculosis incidence and cancer deterioration. This works mainly by activating the patient's immune response. It seems that CD8+ lymphocyte and CD4+ lymphocyte, as well as interferon-gamma (IFN-γ), perform a significant function in tumor antigen identification. Moreover, the increased amount of pro-inflammatory cytokines improves the body's immune response by triggering cancer cell phagocytosis. This vaccination has been licensed as a complement to treat bladder cancer.

Streptococcus pyogenes and lymphangioma: These mediate immune cell sensitization. Triggered cells that invade the neoplasm, inhibit further growth and minimize lymphangioma. Using flow cytometry, studies showed that amounts of lymphocytes and neutrophils, and also macrophages, are quickly increasing on the first day after administration of the suspension. Vascular endothelial growth factor (VEGF), tumor necrosis factor-alpha (TNFα), IL-6, IL-8, and NK cell levels also increased.

**Figure 2:**
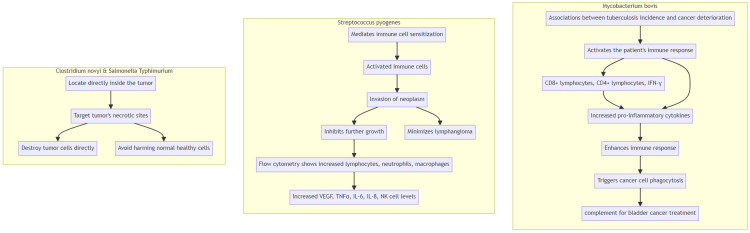
Microbiota as Anticancer Agent

Clostridium novyi and Salmonella enterica serovar Typhimurium: The greatest advantage of using these microorganisms is that they locate directly inside the tumor, in contrast to chemotherapeutics, which spread throughout the body with blood, also destroying normal, healthy cells. Bacteria grow in the necrotic sites of the tumor and may destroy tumor cells directly (Figure 2) [6].

Gut microbiota as a therapeutic target

Modulating gut micro-organisms may enhance the treatment responses to cancer and also reduce toxicity that is caused by treatment. The modification of the gut bacteria can be done via the implantation of fecal microorganisms, changes in diet and lifestyle, prebiotic administration, and different bacterial organisms. In addition, bacteriophages or antibiotic treatments can achieve the targeted modulation of gut microbiota [47].

Diet is one of the factors effective at regulating gut micro-organism's structure. Evolving findings support associations between diet-microbiome and chemotherapy. In addition to food, dietary supplements, postbiotics, probiotics, and synbiotics are also used for the modulation of gut microbiota [47,48].

Studies have shown that mice fed a regime rich in fish oil, protein, and different monosaccharides significantly reduced the population of Pseudomonas aeruginosa. Also, fasting can decrease the side effects of chemotherapy. Fasting has been shown to reduce the vomiting associated with doxorubicin. Therefore, fasting can protect dogs from the adverse effects of irinotecan therapy [48].

Tumor microbiota as a cancer therapy target

Efforts are being made to attack tumor microbiota by modifying gut microbiota to inhibit tumor development and enhance treatments. Antibiotic administration may improve the responses to chemotherapy because the intratumoral microbes are documented to have a negative impact on the treatment response [49]. Systemic antibiotic administration can also affect gut microbiota. Knowing the role and structure of the tumor microbiota leads to targeting tumor-living microbes as a means of specific cancer treatment targets [50].

Tumors can be home to anaerobic bacteria. Tumor microbiota-specific targeting can take benefit of necrotic regions and hypoxic microenvironments of the tumor. For example, the spores of Clostridia that germinate in anoxic tumor sites may produce products that disrupt tumor cell membranes also cause deterioration of solid tumors, activation of the immune system mediated by T cells + CD8 and NKCs, and thus may make a therapeutic approach [50].

Microbiota and chemotherapy

Microbes are used as a vector that contains particular anticancer products and enzymes that are useful in the destruction of cancer cells. The use of microbiota as a vector to transfer an anticancer agent directly into the tumor will significantly reduce the side effects of treatment that are usually accompanied by chemotherapy [28].

Impact of Chemotherapy on Gut Microbiota

Gut microbiota has an impact on the response to cancer therapy, while microbiota is affected by cancer treatment. Chemotherapy may influence various metabolic pathways and has the potential to cause dysbiosis. Antibiotics that are administered during chemotherapy impact microbiota. The use of combined antibiotics during treatment has been shown to have negative consequences on cancer immunotherapy efficacy [47].

Microbiota and Chemo-Resistance

Studies have shown that gut bacteria enhance chemo-resistance. Recognizing the effect of antitumor agents on microbiota may explain mechanisms of chemo-resistance, helping to establish approaches on the way to enhance the effectiveness of therapy. Inflammation and tumor microenvironment play an important role in promoting chemo-resistance. For example, there is a high amount of Fusobacterium in CRC that may facilitate tumor growth associated with poor prognosis [51].

Additional studies revealed that F. nucleatum can protect tumor cells from antitumor agents, which leads to chemotherapy resistance in CRC patients. Co-culturing HCT-116 colon cancer cells with F. nucleatum leads to reduced apoptosis caused by oxaliplatin and 5-fluorouracil (5-FU). Also, F. nucleatum infection can reduce the chemosensitivity of CRC cells to 5-FU by upregulating the expression of baculoviral inhibitor of apoptosis repeat containing 3 (BIRC3), an apoptotic protein inhibitor that prevents apoptosis by directly inhibiting the caspase cascade [52].

Fusobacterium adhesin A (FadA) of F. nucleatum interacts with E-cadherins which in turn activate the signaling of Wnt/β-catenin and may induce proliferation and tumorigenesis. F. nucleatum additionally inhibits the action of T cells plus NK, which limits their ability to combat adenocarcinoma in the colon. Furthermore, mice experiments have shown that antibiotic use suppresses F. nucleatum significantly increases chemotherapy efficacy [28].

Streptomyces may inactivate anthracyclines via NADH dehydrogenase-mediated deglycosylation mechanism. Also, the efficacy of several anticancer drugs based on fluoropyrimidine nucleoside may be impaired via the existence of mycoplasma organisms. All these chemotherapeutic drugs must, therefore, be co-administered with some antibiotics or mycoplasma inhibitors to avoid the drug's inactivation at the tumor site [53].

It is proposed that inflammatory response is an important factor in altering chemo-resistance as well as enhancing metastasis. An essential microorganism involved in the inflammatory condition of the oral cavity is Porphyromonas gingivalis. P. gingivalis is proposed to elicit paclitaxel resistance in oral squamous cell carcinoma through a notch1-dependent mechanism [28].

## Conclusions

Significant evidence now exists, in both humans as well as in laboratory animals, that the commensal microbiota has a significant role in cancer pathogenesis, growth, and therapy response. The impact of microbiota on carcinogenesis could be mediated by affecting the host immune system, influencing the metabolism of many products, and/or changing the cell cycle balance. Therefore, targeting the microbiota could have potentially important beneficial effects on cancer treatment, by using microbiota to enhance the immune system response or as an adjuvant therapy that potentiates other anti-cancer methods, hence, exerting better outcomes.
